# IT Capability, Organisational Learning and Innovation Performance of Firms in Kenya

**DOI:** 10.1007/s13132-021-00886-8

**Published:** 2022-02-24

**Authors:** Chuks Otioma

**Affiliations:** UNU-MERIT: United Nations University-Maastricht Economic and Social Research Institute On Innovation and Technology, Maastricht University, Boschstraat 24, 6211 AX Maastricht, Netherlands

**Keywords:** IT capability, Organisational learning, Innovation, Business value of IT, Kenya, Sub-Saharan Africa

## Abstract

This paper explores the role that IT capability (IT-c) plays in firm innovation performance through the channel of organisational learning (OL) in Kenya. It frames OL in two dimensions: explorative and exploitative OL. The former entails seeking new knowledge, which mainly exists outside the firm’s competence. The latter is an activity or process that builds on existing competence and knowledge in the firm. Using mediation analysis of 481 firms drawn from the World Bank Enterprise Survey (2013) and Innovation Follow-up Survey 2014, it demonstrates that IT-c has a significant effect on innovation performance of firms (capability to simultaneously improve products, processes, organising and market development). It finds that the mediative role of OL in the relationship between IT-c and innovation performance is realised mainly through explorative learning, whilst enabling the firm exploit existing (in-house) knowledge base. The paper puts forward some managerial, policy and further research suggestions.

## Introduction

Information technology (IT)^1^ is changing how businesses are conducted and holds promise for firms. The deployment of IT in business is evident in the shift in business activities towards its use to create value in new ways for the firm and customers (Gobble, 2018). Whilst recognition of the adoption of IT amongst firms is not an issue, the fundamental questions arise in the empirical establishment of the business value of IT and the mechanisms through which it is realised in firms (Kuusisto, 2015). The *business value* or *performance effect of IT* refers to its benefit(s) for firms as may be captured in various ways, for example improvement in product development and process improvement, return on asset or any economic added value for the firm that derives from IT (Bharadwaj, 2000). In the context of this study, the business value of IT in focus is the contribution of IT to “product, process, organisational and marketing improvement”, for which this study applies the term *innovation performance*.

Earlier studies provide evidence for the performance effect of IT (Bharadwaj, 2000; Wade & Hulland, 2004). However, it is insufficient to identify its direct impact on firms, without the complementary resources that account for the impact (Brynjolfsson & Hitt, 2000). Lee et al. (2014) support this position. They argue that there is evidence for the effect of IT on firms, but “we still have limited understanding about the process through which IT contributes to business value” (p. 111). This is rooted in the thinking that IT has little or no significance for firms if not integrated with firms’ resources and processes. An established firm process that accounts for differences in firms’ performance is organisational learning (OL).^2^ Accordingly, Tippins and Sohi (2003) argue that OL could be a missing link in the business value of IT. OL is important because it is a source of competitive advantage. Firms that learn are able to gather information about the market and tend to be more flexible hence can respond to changes in the business environment (Jiménez-Jiménez & Sanz-valle, 2011). Such firms are also able to build on their existing knowledge and modify internal practices to improve product development, process and find new markets. This positions OL as a key mediator of the impact of IT on firms.

The discourse is relevant to the Sub-Saharan African (SSA) context, where the deployment of IT in firms has received attention. Whilst most of the existing literature on Africa recognises the potential of IT in enhancing firms’ communication and business opportunities (Hjort & Poulsen, 2018; Matambalya & Wolf, 2001; Paunov & Rollo, 2015), there still remains a gap for two reasons. First, most of these studies (for example Hjort & Poulsen, 2018; Paunov & Rollo, 2015) focus on the impact of IT adoption or coverage at industry levels and/or aggregated for countries. Second, they do not investigate the mechanism of the IT effect within firms.

The COVID-19 shock provides further justifications for the research and policy importance of exploring the links between IT capability and firm innovation performance in SSA. In the light of the COVID-19 shock and economic lockdown, more economic transactions have shifted online as businesses turn to digital platforms to conduct their activities with potentials to mitigate the negative effects on output (OECD, 2021; Soluk et al., 2021; World Bank, 2021; Kuckertz et al., 2020).

In line with the business value of IT research need identified, the aim of this paper is to explore the relationship between IT capability (IT-c) and firm innovation performance in Kenya, and the role of OL in the relationship. The role of OL in the relationship between IT-c and firm innovation performance is expected to be such that IT enables the firm to improve its OL activities, for example creation and management of knowledge, and facilitation of knowledge sharing across functional units, which in turn enhance the firm’s ability to develop new products, improve process and expand market.

The paper contributes to filling relevant research gaps in several ways. First, it adds to the body of work that investigates the role of IT in firms which are a key agent in its deployment and at which level the effect can be precisely captured (Brynjolfsson & Hitt, 2000). Second, this study extends the empirical support for the argument that IT per se is not sufficient for impact on firm innovation performance but complementary resources in firms provide the mechanisms of impact. Third, it gives insights into the case of Kenya, a key reference in the discourse of IT potential in SSA (World Bank, 2021), but where little evidence is available. Therefore it provides scope for further studies on the firm-level outcomes of IT-c in a developing country’s context. In doing this, it links explorative and exploitative OL to IT-c. The current study opts to analyse exploitative and explorative learning apart in order to identify the specific OL channels of the IT effect. Exploitative learning refers to the process of building on existing competence of the firm; drawing from the firm’s experience and routines whilst explorative learning refers to searching for knowledge that is unfamiliar to the firm, outside its existing competence. OL is elaborated in the literature section drawing from earlier studies (Huber, 1991; March, 1991).

The rest of the paper proceeds as follows. The “Review of Literature” section presents a review of literature, with focus on conceptual clarifications of IT-c and OL. It later presents arguments for the links between IT-c, OL and innovation performance of firms. In the “Methodology” section, the data and methods are described. The “Results and Discussion” section presents the findings of the study and places them in the context of existing studies. The “Conclusions and Reflections” section concludes, with reflections on the study and opportunities for policy and research.

## Review of Literature

### Conceptual Clarifications

#### IT Capability of Firms

Various terms have been applied to define IT capability (IT-c). Bharadwaj (2000) conceptualises IT-c as IT infrastructure (computers, other communication technologies and sharable platforms), human IT (IT skills; technical and managerial) and IT intangibles (corporate culture, customer orientation and environmental orientation). In a related framing, Tippins and Sohi (2003) capture three elements: IT objects (e.g. software and hardware), IT knowledge (knowledge about IT objects) and IT operations (the extent to which IT is applied to activities of firms). Bhatt and Grover (2005) define IT capability in terms of IT infrastructure, IT business experience (an IT group that has business expertise) and IT relationship infrastructure (the extent to which the IT and business units communicate). The authors argue that the possession of IT and business skills amongst the IT group is important for business problem-solving that requires a combination of technical and business expertise. IT relationship infrastructure allows for data acquisition and information sharing as the IT and business groups find solutions to problems. Digital transformation requires a configuration of IT human capital such that digital, analytical and business skills are intertwined. In this development, online marketing experts and data analysts that have IT skills and knowledge of product marketing, for example, take over the function of marketing research and traditional brand/product marketing (Verhoef et al., 2021) whilst business intelligence groups support the development of IT infrastructure (Fink et al., 2017).

Wielgos et al. (2021) present a three-construct framing of digital capability: digital strategy, digital integration and digital control. Digital strategy refers to the extent to which a firm aims to create new forms of value for itself, its customers and partners through a combination of digital technologies. However, customers are the core of digital strategy. Digital integration is the extent to which a firm forms linkages that help to coordinate business processes within and across its boundaries to deliver new forms of value through a combination of digital technologies. This dimension reflects mainly supply chain management activities. Digital control points to the IT-enabled capability to monitor and evaluate procedures and systems in order to maintain and/or adapt the organisational activity to changes. This dimension relates mainly to IT-enabled development, monitoring and assessment of key performance indicators, and market scanning. Freitas (2018) submits that digital capabilities are a combination of skills and digital business process required to develop and mobilise resources to respond to the market and add value to an organisation.

Adapting the studies cited above, the framing of IT in this paper takes into account the recurring themes: IT infrastructure (shared platforms, e.g. website), IT skills (IT use amongst staff and existence of a group of IT specialists) and operations. This is consistent with OECD's (2020) position that firms’ success in the information technology age requires workers with literacy, numeracy and general IT skills alongside IT specialists and complementary competences needed for new organisational forms.

#### Organisational Learning

Huber (1991) recognises four components of OL: knowledge acquisition, distribution, interpretation and organisational memory. Knowledge acquisition comprises information available to the firm, for example, through search around the organisation’s environment. Knowledge distribution is the circulation of knowledge created (or acquired) amongst staff or functional units for improving its operations and decision-making. Information interpretation^3^ refers to the capability of the staff to arrive at a common meaning of information and use it in operations and strategy. Organisational memory is the ability to keep the knowledge cycle: using, reusing and possibly improving the existing knowledge. Organisational memory could be declarative, which refers to facts and events, or procedural, which refers to business routines and procedures (Tippins & Sohi, 2003). Hurley and Hult (1998) see organisational learning as the process of market intelligence gathering and sharing targeted at improving the innovation capability of firms. Put together, OL assumes two broad streams: exploration, which refers to research/information seeking about knowledge not already in the firm, and exploitation, which refers to building on and applying the firm’s familiar competence and knowledge (March, 1991). Do and Mai (2021) conduct a review and submit that Huber’s four-dimensional OL construct is applied in most studies on the links between OL and firm performance whilst other constructs such as climate for learning and experimentation have also been applied in some studies.

This study supports the argument that the two approaches to organisational learning (exploration and exploitation) in firms provide routes to realising the effect of IT. This is because the activities that characterise OL such as scanning for knowledge (research), trying out new ideas (experimentation) and retrieving stored knowledge represent the areas in which IT can help the firm improve innovation performance. For the purpose of operationalising these concepts in this study, explorative OL is framed in two components: *research-oriented learning* and *experimentation-oriented learning*. Research-oriented organisational learning reflects activities that are concerned with knowledge acquisition which could be by way of R&D and/or market research whilst experimentation-oriented learning refers to activities or practices related to trying new ideas and technologies. This takes into account the reasoning that explorative learning is characterised by such things as search, experimentation and risk-taking (March, 1991). The rationale for sub-dividing explorative learning also takes into account nature of data and analysis conducted in this study; in that, the firms studied tend to learn in more diverse ways in terms of explorative learning than exploitative learning. Therefore, exploitative learning is used in one dimension.

### Hypotheses

#### IT Capability and Innovation Performance

IT capability influences firm innovation performance as it promotes shift towards IT-enabled business process and operations. Aral and Weill (2007) point out that IT benefits the firm, for example, as IT resources that support internal management, reporting and information sharing enhance decision making.

In a study of firms drawn from 11 OECD countries, Spiezia (2011) finds that firms with higher intensity of IT use are more innovative, especially in product and marketing innovation. This is consistent with Arvanitis and Loukis' (2016) study of 743 hospitals in 18 European countries in which IT applications such as use in patient administration and medical record have a positive effect on product and process innovation. In a study of 288 firms in Mexico, Cuevas-vargas et al. (2016) demonstrate that there is a positive and significant relationship between IT applications and product, process and organisational innovation. Based on a rich dataset of over 2 million SMEs drawn from 24 European countries, Scuotto et al. (2021) evaluate the effect of digital capabilities, with focus on IT skills (information, communication and software skills). They find that digital capabilities positively influence SME innovation performance (ongoing innovation activities, prototypes and innovation developed), which helps the firms increase sales and employment growth. Blichfeldt and Faullant (2021) conduct a study of manufacturing firms in process industry and conclude that digital technology adoption is positively related to product/service innovation. Matarazzo et al. (2021) present a case study of Italian SMEs and confirm that digital capability helps them in business model development, creation of new channels and delivery of value to customers.

The preceding literature leads to the following:

- *Hypothesis 1(H1): IT-c is positively related to innovation performance of firms.*

#### IT Capability and Organisational Learning

IT capability is linked to explorative OL, partly in form of research (which may be basic or market-related research). IT plays a role in research-oriented explorative learning in form of acquisition of new knowledge and market sensing. Market knowledge acquisition is a channel for firms to collect and integrate useful information from and about market actors. Zhou and Li (2012) point out that a firm’s knowledge base alone is not sufficient to succeed in product development; therefore, the firm must collect and use external resources such as market information. Not only does market knowledge help the firm in product development but also enables it to search for information about new markets.

Lu and Ramamurthy (2011) present two ways in which firms monitor and respond to changes in the market: market capitalising agility and operational adjustment. Whilst the former entails monitoring the market to improve product to satisfy customer needs the latter means that the firm must continuously refine internal processes to meet such needs. They therefore posit that IT serves as a capability to handle the volume and variety of information that is required to monitor and respond to the market. IT enables scanning of the market signals and management of internal information to make decisions to improve product, process and seize new market opportunities (Abbate et al., 2021; Ravichandran, 2018). In some cases, market sensing informs the firm’s decision in setting the trend for new demands by introducing products that are at the edge of technology. This is consistent with Cai et al.'s (2017) position that product development is a knowledge-intensive activity, hence requires IT in the acquisition and integration of information that could be helpful for innovative initiatives, for example, knowledge about consumer preferences and technical knowledge about product engineering. Based on a case study of a Steel Plant in India, Agrawal et al. (2020) demonstrate that the managers’ integration of IT with knowledge management to update its processes and adapt to the changing market conditions has helped the company maintain a competitive standing in the industry.

The other area of explorative learning where IT-c enhances OL is experimentation. Experimentation involves the identification of and trial of new ideas and/or technologies. Hampel et al. (2020) argue that experimentation is based on an iterative process which incorporates feedback into product development. The process which entails formulating product ideas, testing, adjusting and validating helps firms improve product development. This agrees with Bocken and Snihur's (2020) thinking that experimentation provides opportunity to engage customers and other actors in the innovation process. The iterative process of experimentation allows the firm to generate novel and impactful ideas.

The value of IT-c for experimentation is linked to the capability of digital platforms to facilitate interaction amongst teams who engage in the proposition, clarification of and feedback on new ideas (Benitez et al., 2018), and enhancement of flexibility or informal socialisation that facilitates heterogeneous knowledge contribution (Cenamor et al., 2019).

IT capability is also linked to exploitative OL. Organisational memory enhancement is one area that highlights the role of IT in exploitative OL. IT is linked to organisational memory through its role in knowledge coding and transfer (Grant, 1996), as well as retention, recovery and re-use in the innovation process (Antunes & Pinheiro, 2020; Gonzalez & Melo, 2018). Jackson (2010) points out that database, for example, allows the storage of information about repeated processes in firms whilst intranet helps hold documents that could contain written guides, designs and initial versions of products that can be shared amongst staff for further development. This is in addition to multi-media applications (e.g., Skype, Zoom and other online conferencing applications) that enable individuals to communicate some knowledge that is difficult to code and transfer.

In line with the preceding literature this study poses that:

- *Hypothesis 2a (H2a): IT-c is positively related to research-oriented organisational learning of firms.*
- *Hypothesis 2b (H2b): IT-c is positively related to experimentation-oriented organisational learning of firms.*
- *Hypothesis 2c (H2c): IT-c is positively related to exploitation of existing knowledge/technology in firms.*

#### Organisational Learning and Innovation Performance

Explorative learning in form of knowledge acquisition (through research) and/or experimentation can be demonstrated to enhance innovation performance of firms. Some studies demonstrate that internal R&D influences firm innovation performance. For example, Zhang and Tang (2017) demonstrate that knowledge sharing amongst the internal R&D team enhances firms’ innovation performance, and that the effect is strengthened when the composition of internal R&D employees is heterogeneous, enabling them to promote diverse ideas. Dolores (2016), in a study of Spanish manufacturing firms for the period 2006–2012, demonstrates that conducting internal (basic) research affects a firm’s propensity to innovate.

In this paper, internal R&D efforts do not necessarily mean that the knowledge generated originates from the firm; it more accurately indicates the practice of using a firm’s internal R&D team (in contrast to outsourcing) in knowledge acquisition. This point is instructive in a context where knowledge sourcing could be done by the internal R&D team yet mainly in form of searching for available (external) knowledge and technological trends to mimic. For example, in the related case of South Africa, previous studies (Chan & Oerlemans, 2011; Oerlemans & Pretorius, 2006) find that explorative route to learning in form of scanning for knowledge in other firms, which is then mimicked, is the dominant source of knowledge to boost innovation performance of firms, where internal knowledge base is weak.

Research outsourced to external consultants, particularly to conduct market research (as is the focus in the case of Kenya) augments firm’s internal knowledge creation or acquisition efforts. Market information scanning, also known as market sensing, enables firms learn about consumers, suppliers and competitors, and adapt to trends. In a study of 168 firms in Indonesia, Ardyan (2015) finds that market sensing capability positively affects speed to market and firm innovation.

As regards experimentation and innovation performance, the former allows a level of autonomy to try new initiatives in solving existing or new problems. This promotes the development of new competence and creation of new knowledge (Gonzalez & Melo, 2018). Empirical studies show a positive relationship between experimentation and innovation performance of firms. For example, Bouwman et al. (2018) find that competitive environment intensifies business model experimentation which then influences innovativeness. Chang et al. (2012) demonstrate, in the case of 500 Taiwanese firms, that experimentation is positively associated with radical innovation, which the authors define as breakthrough product or process (new-to-the world; new-to-the generation). Using a case study of clinics, Stan and Vermeulen (2013) report that experimentation helps organisations learn through encouraging staff to handle new and difficult tasks. Knowledge from difficult cases aids handling of relatively simple ones whilst the innovative teams deepen understanding of problems and new solutions.

On the other hand, exploitation is related innovation performance of firms by way of building on existing knowledge. This is rooted in the thought that history matters and that firms’ opportunities to learn and innovate tend to be local and cumulative, closely connected to previous activities and knowledge, in a manner that requires gradual changes to routines (Lundvall, 2007; Teece & Pisano, 1994). When firms exploit their knowledge base they gain efficiency, reduce search cost, minimise the risk associated with trials and speed up implementation of innovative initiatives (March, 1991; Stan & Vermeulen, 2013). These happen because much is known in-house about the product, process and market to which they seek improvement. Therefore exploitative learning enables firms to retrieve and deploy their organisational memory (the knowledge stored and incorporated in their practices and staff) for product and process refinement, and market development.

Previous knowledge forms the foundation for and determines the ease of absorbing new knowledge (Cohen & Levinthal, 1990; Grant, 1996). A firm is a learning organisation not only because it is able to create, acquire and transfer new knowledge and modify its process in light of such new knowledge but also because it is able to draw from its experience (Garvin, 1993; March, 1991; Nevis et al., 1995). Recent empirical studies show that firms’ use of existing knowledge asset has a positive effect on their performance, measured by returns on asset (Cho, 2020) and product innovation (Hecker & Ganter, 2014).

The preceding arguments lead to the following set of hypotheses:

- *Hypothesis 3a (H3a): Research-oriented organisational learning is positively related to innovation performance of firms.*
- *Hypothesis 3b, (H3b): Experimentation-oriented organisational learning positively related to innovation performance of firms.*
- *Hypothesis 3c (H3c): Exploitation of existing knowledge/technology in firms is positively related to innovation performance of firms.*

#### IT Capability and Innovation Performance: the Role of Organisational Learning

Whilst IT in itself does not constitute a source of competitive advantage for the firm, since it is readily available and easily acquired, its integration with OL capabilities such as routines and knowledge repositories accounts for the firm’s distinctiveness and improved performance, in contrast to other firms (Levallet & Chan, 2016; Tippins & Sohi, 2003). Usai et al. (2021) analyse firm data across European countries and find that IT usage has little direct effect on innovation performance. They argue that the low direct effect is due to the ease of acquisition and replication which makes IT weak for competitive advantage of firms. Although not tested, the authors suggest that the role of IT is subsumed in firms’ processes and resources. This corroborates Soluk and Kammerlander’s (2021) proposition that the deployment of IT requires building up technical infrastructure and learning through reorganising routines, and assimilating and commercialising new information. Therefore explorative and exploitative learning activities could serve as mechanisms for realising the gains of IT-c.

A firm that conducts research to sense the market and finds new ways to improve production and service delivery can enhance its innovation performance if its activities are IT-enabled. IT capability enhances the ability of firms to sense the market and then develop products that are aligned with market demand (Rialti et al., 2019; Sambamurthy et al., 2003). It also enables the firm to incorporate customer knowledge into production process and improve responsiveness to customers’ need (Braojos et al., 2019). Incorporating customer’s feedback is part of the market research process that helps at the stage of product development, improvement and customer satisfaction. Tippins and Sohi (2003) demonstrate that IT competence influences OL which then leads to improvement in targeted product development and customer satisfaction.

Considering that IT enhances the trial of new ideas then experimentation could serve as a channel for realising the effect of IT in firms. Koning et al. (2020) show that experimenting with new products, through engagement with customers in testing product design options, improves product features amongst firms that adopt the experimental approach. The authors argue that IT platforms have improved the efficiency of experimentation through the ease of interacting with users in testing new ideas. This is consistent with other studies (D’Adderio, 2001; Thomke, 2003; Thomke et al., 1998) that advances in IT have transformed the use of experimentation in how products are developed, especially when firms apply computer simulations to test new product designs or when IT platforms enable iterations and integration of knowledge for prototypes. Benitez et al. (2018) show that IT resources are positively associated with business experimentation and flexibility (adapting to changing business environment), which in turn boost operational competence and creation of new products.

In the context of exploitative OL, given that organisational memory is at the root of exploitative OL, IT-c helps firms in this area through the volume and ease of information retention, recovery, sharing and (re-)use. The capability of IT to ease the firm’s storage, sharing and (re-) use of knowledge enables it to innovate and improve its competitive advantage (Jackson, 2010). With these attributes of IT in organisational memory enhancement, it can serve as an instrument for improving knowledge exploitation since, according to earlier studies (March, 1991; Stan & Vermeulen, 2013), exploitative learning draws significantly from firm’s experience.

Accordingly, this study argues that:

- *Hypothesis 4a (H4a): Research-oriented organisational learning mediates the relationship between IT-c and innovation performance of firms.*
- *Hypothesis 4b, (H4b): Experimentation-oriented organisational learning in firms mediates the relationship between IT-c and innovation performance of firms.*
- *Hypothesis 4c (H4c): Exploitation of existing knowledge/technology in firms mediates the relationship between IT-c and innovation performance of firms.*

## Methodology

### Model Specification

Following previous methodological frameworks (Arts & Veugelers, 2020; Baron & Kenny, 1986; Hicks & Tingley, 2011), mediation analysis was applied. First, the direct effect of IT-c on innovation performance of firms is estimated in Eq. (1). Second, the effect of IT-c on the mediator, OL, is tested in Eq. (2). Equation (2) is stated in general for OL (mediators) to avoid duplication. However, the individual mediators (research-oriented, experimentation-oriented and exploitative OL) are substituted for in the operationalisation as shown in all the relevant tables. The last model re-estimates the effect of IT-c on firm innovation performance controlling for OL. The procedure is based on three models estimated with OLS regression as follows:

1 $$Innovation\;performance=\alpha_0+\alpha_1\;IT\;capability+\delta X_i+\varepsilon_{i}$$

2 $$Organizational\;learning=\beta_0+\beta_1\;IT\;capability+\delta X_i+\varepsilon_{i2}$$

3 $$\begin{aligned}Innovation\; performance=&\gamma_0\; +\; \gamma_1\; IT\; capability+\gamma_2+research\\ &-oriented\; organisational\; learning+Y_3\; experimentation\\ &-oriented\; organisational\; learning+Y_4\; exploitative\; organisational\; learning\\ &+\delta X_i+\varepsilon_{i3}\end{aligned}$$

where *X*_*i*_ is a set of control variables in the model.

Three conditions must hold for mediation analysis to be valid:

- Condition 1: The independent variable must significantly affect the dependent variable.
- Condition 2: The independent variable must significantly affect the mediator.
- Condition 3: The effect of the independent variable on the dependent variable must weaken or disappear when the mediator is controlled for.

When the conditions for mediation are met in the system of equations, either of the two following possibilities applies: the existence of partial mediation, if α_1_ > γ_1_ or full mediation, if γ_1_ = 0. In the case of full mediation, which rarely occurs, what is taken into account is not the statistical significance of the effect of the independent variable after controlling for the mediator(s) but the coefficient (Baron & Kenny, 1986; Kenny, 2018; Mehmetoglu, 2018).

### Data

This study uses the World Bank Enterprise Survey (WBES) and the World Bank Innovation Follow-Up Survey (WBIFS) in Kenya, covering 2010–2012, reported in 2014. WBES collects data on aspects of institutions such as political stability, tax administration and corruption and firm characteristics. The WBIFS collects innovation-related data on such items as knowledge sourcing, organisational structure and nature of innovation in firms. WBES/WBIFS targets respondents who are the owners or top managers of the businesses surveyed. It covers firms in manufacturing and services, and is representative of firms in non-agricultural sector. These are important for the key aspect of this study, IT capability of firms, which should be easier to find in secondary and tertiary activities in SSA. The survey is stratified according to firm size, sector and location. Given that WBIFS draws sample from WBES (up to 75%), this study merges both modules using the firm identification number. The number of firms, after merger and cleaning, was 781. This reduced to 481 when all the variables of interest were used in the analysis.

### Measures

#### Dependent Variable

The dependent variable (*innovation performance*) considered innovation across all dimensions: product, process, organisational and marketing. The WBES/WBIFS survey follows the definition that “an innovation is the implementation of a new or significantly improved product (good or service), or process, a new marketing method or a new organisational method in business practices, workplace organisation or external relations” (OECD/Eurostat, 2005, p.46).^4^ What underscores innovation is that the new product must have been introduced to the market whilst the process, marketing and organisational methods must have been put into actual use. The paper measured innovation performance of firms as a ratio of the number of dimensions in which a firm has innovated to the number of dimensions possible. The result is then multiplied by 100, to standardise the score in percentage. This aligns with Camisón-Haba et al. (2018) who capture innovation based on whether firms have innovated across dimensions during the years in focus. It is based on the thought that well-performing firms have a mix of innovation across dimensions.

#### Independent Variable

To capture IT-c, this study used principal component analysis (PCA) to extract a latent variable derived from IT use and related activities in firms in WBES/WBIFS. The original items cover questions including whether the firm has purchased software, has a website, engages in online sales and payments and has staff allocated entirely to IT (see Appendix, A1). The original items were binary variables, except computer use (percentage of staff that use computer regularly). In line with the conceptual framing presented in the literature section, the elements of IT objects, skills and operations were considered.

Using a threshold of eigenvalue of 1, the PCA returned three components, explaining 50% of the variability in the data. This means that there are three latent variables that reflect IT and related activities in the firms. Component 1 loads highly on variables that indicate online sales and purchase, marketing of products and to a less extent inventory. This reflects the aspect of e-commerce. Component 2 loads highly on uses of mobile money. The third component has relatively higher loadings on website, computer use amongst staff and, to a less extent, availability of an IT department and emailing. This component reflects the aspect of shared platform, IT skills and information sharing, thus indicates online presence and capability to implement digital transformation in the firm.

Only the third latent variable was applied to measure IT-c in this analysis. The reason is that the two other components show some of the ways IT is being used but do not satisfy the condition for mediation analysis, in line with literature (Baron & Kenny, 1986; Kenny, 2018). Neither of them has significant and consistent effect on OL and innovation performance of firms in this study.

#### Mediating Variable

Organisational learning is the mediator. The original items related to OL were in the binary form in WBES/FIS. The survey asked whether a firm conducted internal R&D, used the service of a market research consultancy, provided training to staff for innovative products, encouraged staff to try new ideas and whether the main innovative product or process is based on existing technology or process already in use by the firm.

PCA was applied to derive OL measures from loadings on the original variables (see Appendix, A2). PCA yielded three components based on a threshold of eigenvalue of 1. These explain 58% of the variability in the OL data. Component 1 loads highly on internal R&D, market research and innovation training. Therefore this component was labelled *research-oriented organisational learning*. The second component loads highly on variables that indicate that innovation is based on existing design/technology in the firm. This component was labelled *exploitative organisational learning*. The third component loads highly on foreign technology licence and encouraging staff to try new ideas. This component was labelled *experimentation*. It indicates experimentation-oriented explorative OL. Accordingly, the components were used as latent variables in the regression analysis, instead of the individual OL variables. First, this helps because PCA ensures that multi-mediator analysis has latent variables that reduce the risk of high correlations amongst mediators (Zhao et al., 2018). Second, they help to reference the aspects of OL, when necessary, given that firms learn in explorative and exploitative ways as argued in the literature section. The OL measurement is grounded in literature. For example, Beyene et al. (2016) capture OL in Ethiopian firms based on information acquisition, dissemination and interpretation. Appelbaum and Reichart (1998) and Chiva et al. (2007) propose encouraging and trying new ideas as an indicator of experimentation.

#### Control Variables

The study controls for firm age which it measured as the difference between when the firm was established and the year the survey was conducted, 2013. Large and medium firms (as defined in Table 1) were taken into account as they are expected to outperform small firms. Two studies on African firms find a positive relationship between firm size and firm performance (Beyene et al., 2016; Biesebrieck, 2005). In line with the reasoning that firms organised as a corporation could be more innovative than those that are not (Barasa et al., 2017), this study controls for legal status. The legal status of the firm takes the value “1” if the firm is organised as a corporation. The control for managerial experience takes a dummy 1 if the top manager has at least 10 years of experience in the industry in which the firm operates. This follows the rationale of 10-year minimum for highly experienced managers in previous studies (Ayyagari & Demirg, 2011; Barasa et al., 2017). Managerial experience reflects the manager’s capability in terms of professional recognition, decision-making and training that could be relevant to the job and firm innovation (Camisón-Haba et al., 2018). This study controls for the firm’s use of a different input in its main innovative product compared with its other products. This indicates varied inputs for innovation, which might be correlated with innovation performance of the firm. To take industry differences into account, the value 1 was assigned to a firm if it belongs to an industry designated high-digital-intensive (as defined in Table 1). Chae et al. (2018) confirm that IT has varied uses and levels of impact according to the firm’s industry.

**Table 1:** Description of variables in the analysis

Variable	Description
1. Innovation performance	The ratio of the number of dimensions in which the firm has innovated to all dimensions of innovation
2. IT capability	Latent variable obtained from principal component analysis for a standardised measure that reflects emailing, web activities and digital skills amongst staff of the firm
3. Research-oriented learning	Latent variable obtained from principal component analysis for a standardised measure that reflects firms’ organisational learning through research
4. Experimentation	Latent variable obtained from principal component analysis for a standardised measure that reflects firms’ organisational learning through trying new ideas and technologies
5. Technology exploitation	Latent variable obtained from principal component analysis for a standardised measure that reflects firms’ organisational learning through adapting its existing designs, processes and technologies
6. Age	The number of years the establishment has been in operation until 2013, when the survey was conducted
7. Large	Binary: 1 is assigned to large firms (number of employees is 100 or more), otherwise 0
8. Medium	Binary: 1 assigned to medium firms (20–99 employees), otherwise 0
9. Legal status	Binary: 1 assigned if a firm is organised as a corporation (one with publicly traded shares and shareholding with non-traded or privately traded shares), otherwise 0
10. Managerial experience	Binary: 1 assigned if the top manager of the firm has at least 10 years of experience in the industry in which the firm operates, otherwise 0
11. Varied inputs	Binary: 1 assigned if a firm has used an input(s) for its main innovative product, different from its other products, otherwise 0
12. Digital industry^*^	Binary: 1 is assigned if the firm belongs to precision instrument, transport machines or IT industry, otherwise 0
13. Main city	Binary: 1 is assigned if a firm is located in Nairobi or Mombasa, otherwise 0
14. External finance	The sum of the proportions (percentages) of working capital financed by various external sources to the firms in the last financial year
15. Institutional burden	Perception scores of the severity of institutional environment of the firm. A score of 1 is assigned to a firm that reports that any of the six dimensions of institutions was at least severe: political instability, high tax rate, weakness in tax administration, prolonged licensing, payment of bribe to public officials and weak laws. The ratio of the number of dimensions reported to be severe to all possible dimensions is calculated and scaled to 10
16. Informal competition	Binary: 1 assigned if the firm competes with unregistered or informal firms, otherwise 0
17. Respondent’s experience	The (length of) time the main respondent in the survey has worked with the firm under consideration

^*^This classification adapts Calvino et al. (2018): Taxonomy of digital intensive sectors

To control for the metropolitan effect on innovation, a value 1 was assigned to a firm if located in Nairobi or Mombasa. Large cities tend to be associated with the co-presence of incumbent firms and start-ups that enable knowledge spillover (Feldman & Kogler, 2010; Quian, 2018), and associated with resources and enabling institutions. It also controls for external finance. The measure of firms’ access to external finance is the sum of the proportions (percentages) of working capital financed by various external sources to the firms in the last financial year. Firms need finance to make investments in technologies and access markets that promise higher returns (Lazonick, 2006). This study recognises that activities of informal firms mount pressure on formal firms to innovate (Perez et al., 2019). The firm was assigned 1 if it reported that it faced competition from informal firms.

The control for institutional burden was constructed from six items in WBES/WBIFS. This captures how much each item poses an obstacle to business operations, on a 5-point scale, from 0 (no obstacles) to 4 (very severe obstacle). The original items relate to rule of law (questions on the degree of obstacles posed by courts, political instability and corruption), and regulation (obstacles posed by tax rate, tax administration and processing business permits). These measures were rescaled to the value 1 if the respondents reported an item to be at least a major obstacle, that is major or very severe, otherwise “0” (no obstacle to moderate obstacle). The score for each firm was calculated as the ratio of the number of items in which it reported “major or severe obstacle” to all obstacles considered. Firms that report higher institutional burden face difficulties in conducting business; therefore it could be negatively related to innovation. Regional institutional quality complements firms’ internal resources to drive innovation (Barasa et al., 2017), and the effect of institutions on innovation capability is more important in countries that have more room for improvements (Kafka et al., 2021), as is the case of Kenya. This study controls for the main respondent’s experience, with an expectation that a respondent with longer experience (defined in Table 1) in the firm would be so used to it that he/she would see less change, possibly critical of innovation. Therefore he/she tends to be less likely to report a product, process, organising or marketing as innovative.

## Results and Discussion

### Results

#### Descriptive Statistics

Table 2 shows the descriptive statistics and correlations of variables in the analysis. IT-c and OL (research, experimentation and technology exploitation) have the highest correlation with innovation performance. This is an initial indication that IT-c is positively associated with OL. The correlations between the mediators and the IT-c indicate that the two exploration-based learning approaches (research-oriented learning and experimentation) have the highest correlation with IT-c, followed by exploitative learning (technology exploitation). Considered together, the low correlations between variables mean that there should be minor or no concern for multicollinearity. Multicollinearity arises where the explanatory variables are highly correlated. However, to further check for possible multicollinearity, variance inflation factors (VIFs) were estimated, such that the variables that violate the tolerance requirement would be excluded from the analysis. The estimated VIFs range from 1.03 to 1.62, with a mean of 1.22. None of the explanatory variables had a VIF that exceeded a limit of 3.0 (Picón et al., 2014).

**Table 2:** Summary statistics of variables

Variable	Mean	SD	Min	Max	VIF	1	2	3	4	5	6	7	8	9	10	11	12	13	14	15	16	17
Innovation performance	60.88	36.40	0	100		1.00																
Age	22.93	18.07	1	107	1.37	0.12	1.00															
Large	0 0.26	0.44	0	1	1.62	0.12	0.21	1.00														
Medium	0.35	0.48	0	1	1.32	0.10	− 0.03	− 0.45	1.00													
Legal status	0 0.18	0.39	0	1	1.09	0.07	0.19	0.12	− 0.03	1.00												
Managerial experience	0.78	0.41	0	1	1.13	0.07	0.18	0.10	− 0.06	0.08	1.00											
Varied inputs	0.19	0.39	0	1	1.25	0.18	− 0.00	0.20	− 0.04	0.02	0.03	1.00										
Digital industry	0.04	0.20	0	1	1.05	0.06	− 0.07	− 0.06	0.04	− 0.07	0.03	0.05	1.00									
Main city	0.63	0.48	0	1		0.10	− 0.09	0.14	− 0.01	− 0.07	0.05	0.17	0.11	1.00								
External finance	36.99	36.47	0	100	1.05	0.06	0.06	0.01	− 0.00	0.08	0.07	0.00	− 0.02	− 0.02	1.00							
Institutional burden	2.14	2.55	0	10	1.03	0.03	0.04	0.01	0.04	0.07	− 0.00	− 0.02	0.05	0.00	− 0.02	1.00						
Informal competition	0.55	0.50	0	1	1.04	0.06	− 0.03	− 0.07	0.02	− 0.03	0.05	− 0.01	0.08	− 0.02	0.04	0.07	1.00					
Respondent’s experience	11.53	9.17	2	55	1.30	− 0.03	0.35	− 0.06	0.00	0.00	0.28	− 0.06	− 0.05	0.01	0.07	0.04	− 0.01	1.00				
Org. learning (research)	− 7.66e − 9	1.38	− 1.12	4.68	1.41	0.33	0.13	0.22	− 0.05	0.12	0.03	0.39	0.08	0.09	0.02	0.05	− 0.02	− 0.06	1.00			
Org. learning (experiment.)	− 1.02e − 8	1.06	− 1.96	3.00	1.19	0.37	0.21	0.19	0.05	0.09	0.09	0.10	0.03	− 0.04	0.12	0.04	− 0.10	− 0.03	0.17	1.00		
Org. learning (tech exploit.)	− 4.35e − 9	1.27	− 1.14	4.10	1.16	0.23	0.03	0.10	0.06	0.10	0.02	0.20	0.04	0.08	0.05	0.09	0.06	− 0.01	0.30	0.11	1.00	
IT-c	5.42e − 10	1.49	− 3.20	3.11	1.42	0.22	− 0.01	0.31	− 0.02	0.07	0.04	0.28	0.11	0.23	− 0.09	0.00	− 0.01	− 0.13	0.37	0.23	0.22	1.00

*n* = 481 (correlation, VIF), *n* = 545 (IT-c), *n* = 719 (respondent’s experience), *n* = 730 (external finance), *n* = 755 (age), *n* = 781 (others). Mean VIF = 1.22

#### Confirmatory Regression Analysis

The paper argues that innovation performance of firms is positively associated with IT-c. The framing of the relationship between them is such that OL variables serve as a mechanism through which IT-c influences innovation performance of firms. Table 3 presents the initial test. In Table 3 (column 1) control variables were used to check their influence on the model and how the inclusion of IT-c and OL variables later would affect innovation performance. In column 2 (or hypothesis 1, H1), the argument about the effect of IT-c on innovation performance was supported. The model confirmed that the higher the orientation of firm towards IT in its business activities, the higher the firm’s level of innovation performance indicated by introducing innovation across dimensions. This relationship is significant at *b* = 3.59, *p* < 0.01.

**Table 3:** Regression of innovation performance on IT-c

	(1)	(2)
Age	0.142 (0.085)	0.225* (0.097)
Large	6.740 (3.792)	6.711 (4.491)
Medium	10.89*** (3.230)	11.04** (3.693)
Legal status	2.392 (3.651)	3.068 (4.372)
Managerial experience	2.876 (3.702)	4.290 (4.251)
Varied inputs	11.39** (3.519)	8.934* (3.717)
Digital industry	11.550 (6.485)	6.858 (7.651)
Main city	7.941** (2.969)	3.223 (3.415)
External finance	0.069 (0.037)	0.061 (0.043)
Institutional burden	− 0.238 (0.548)	0.172 (0.661)
Informal competition	6.093* (2.777)	4.680 (3.172)
Respondent’s experience	− 0.238 (0.161)	− 0.211 (0.188)
IT-c		3.589** (1.212)
Constant	39.00*** (4.579)	39.58*** (5.362)
*n*	662	481
Adj. *R*^2^	0.06	0.09

Standard errors (SE) in parentheses: *p < 0.05, **p < 0.01, ***p < 0.001

The key argument is that IT-c can influence firms’ innovation performance through enabling or improving OL in form of research-oriented activities, experimentation with and sharing new ideas and effective use of knowledge in the firm, for example, in form of ease of recording and retrieval (organisational memory). If this is the case, then IT-c must be positively (significantly) associated with OL variables in the regression model.

Accordingly, Table 4 (column 1) tests whether IT-c relates positively to research-oriented OL (H2a), as evidence for the latter to function as a mediator between IT-c and innovation performance of firms. The results show that IT-c is indeed positively and significantly associated with research-oriented OL; hence, the latter is a possible mediator. In the next column, IT-c is positively related to experimentation, which is also a variable of knowledge exploration (in support of hypothesis H2b). Table 4 (column 3) shows that IT-c is positively related to technology exploitation (H2c).

**Table 4:** Regression of organisational learning on IT-c

	(1) Organisational learning (research)	(2) Organisational learning (experimentation)	(3) Organisational learning (exploitation)
Age	0.011** (0.004)	0.011*** (0.003)	− 0.000 (0.004)
Large	0.126 (0.173)	0.311* (0.131)	0.208 (0.185)
Medium	− 0.057 (0.142)	0.272* (0.108)	0.275 (0.152)
Legal status	0.251 (0.168)	0.021 (0.128)	0.323 (0.180)
Managerial experience	− 0.042 (0.164)	0.191 (0.124)	− 0.053 (0.175)
Varied inputs	1.028*** (0.143)	0.088 (0.109)	0.491** (0.153)
Digital industry	0.408 (0.295)	0.211 (0.224)	0.118 (0.315)
Main city	− 0.041 (0.131)	− 0.206* (0.100)	0.0443 (0.141)
External finance	0.001 (0.002)	0.004** (0.001)	0.002 (0.002)
Institutional burden	0.010 (0.026)	0.018 (0.019)	0.050 (0.027)
Informal competition	− 0.037 (0.122)	− 0.213* (0.0927)	0.184 (0.131)
Respondent’s experience	− 0.010 (0.007)	− 0.010 (0.006)	0.003 (0.008)
IT-c	0.275*** (0.047)	0.147*** (0.035)	0.154** (0.050)
Constant	− 0.137 (0.206)	− 0.452** (0.157)	− 0.333 (0.221)
*n*	481	481	481
Adj. *R*^2^	0.24	0.14	0.07

SE in parentheses: *p < 0.05, **p < 0.01, ***p < 0.001

In further confirmation of the mediative role of OL, innovation performance was regressed on OL dimensions, controlling for IT-c. Controlling for the independent variable in confirming the influence of the mediator on the dependent variable is important since the independent variable could significantly affect both to the extent that the possible mediator loses its significance (Kenny, 2018). Table 5 shows that research-, experimentation-oriented and technology exploitation OL variables are positively related to innovation performance of firms (H3a-3c) in column 2-4. These results are statistically significant at (*b* = 6.22, *p* < 0.001), (*b* = 10.67, *p* < 0.001) and (*b* = 3.82, *p* < 0.001) for research-, experimentation-oriented and technology exploitation OL respectively. This provides the statistical support for OL to serve as a mechanism through which IT-c contributes to firms’ innovation performance.

**Table 5:** Innovation performance on IT-c and organisational learning

	(1)	(2)	(3)	(4)	(5)
Age	0.225* (0.097)	0.156 (0.095)	0.108 (0.094)	0.226* (0.096)	0.0507 (0.091)
Large	6.711 (4.491)	5.931 (4.367)	3.396 (4.297)	5.917 (4.446)	2.288 (4.157)
Medium	11.04** (3.693)	11.390** (3.591)	8.137* (3.537)	9.989** (3.664)	7.845* (3.430)
Legal status	3.068 (4.372)	1.506 (4.260)	2.847 (4.159)	1.835 (4.337)	0.669 (4.038)
Managerial experience	4.290 (4.251)	4.550 (4.132)	2.254 (4.053)	4.494 (4.203)	2.677 (3.917)
Varied inputs	8.934* (3.717)	2.542 (3.807)	7.999* (3.538)	7.062 (3.715)	1.182 (3.612)
Digital industry	6.858 (7.651)	4.320 (7.452)	4.605 (7.284)	6.407 (7.565)	2.148 (7.052)
Main city	3.223 (3.415)	3.479 (3.320)	5.422 (3.263)	3.054 (3.377)	5.468 (3.154)
External finance	0.061 (0.043)	0.052 (0.041)	0.021 (0.041)	0.053 (0.042)	0.010 (0.040)
Institutional burden	0.172 (0.661)	− 0.012 (0.644)	− 0.023 (0.630)	− 0.019 (0.656)	− 0.305 (0.611)
Informal competition	4.680 (3.172)	4.910 (3.084)	6.949* (3.034)	3.977 (3.143)	6.619* (2.940)
Respondent’s experience	− 0.211 (0.188)	− 0.156 (0.183)	− 0.103 (0.180)	− 0.221 (0.186)	− 0.065 (0.174)
IT-c	3.589** (1.212)	1.881 (1.221)	2.023 (1.173)	3.001* (1.210)	0.182 (1.176)
Org. learning (research)		6.216*** (1.168)			5.457*** (1.128)
Org. learning (experimentation)			10.67*** (1.506)		10.35*** (1.457)
Org. learning (exploitation)				3.815*** (1.110)	2.517* (1.054)
Constant	39.58*** (5.362)	40.43*** (5.214)	44.40*** (5.145)	40.85*** (5.314)	45.84*** (4.982)
*n*	481	481	481	481	481
Adj. *R*^2^	0.09	0.14	0.17	0.11	0.23

SE in parentheses: *p < 0.05, **p < 0.01, ***p < 0.001

In Table 5 (column 5), IT-c and mediators are included to re-estimate the effect of IT-c on innovation performance of firms. It shows that the effect of IT-c on innovation performance, as initially observed in an earlier model (Table 3, column 2, replicated in Table 5, column 1) weakens (from *b* = 3.59, *p* < 0.01) (in Table 5, column 1) to (*b* = 0.18, with *p* insignificant) (in Table 5, column 5), when controlling for OL (mediators). This indicates that a significant part of the initial effect of IT-c on innovation performance has been taken up by OL variables which remain significant in the full model (Table 5, column 5). In line with the conditions for mediation outlined in the method, it confirms that OL is a partial mediator of the relationship between IT-c and innovation performance of firms (H4a–4c). A more detailed examination of the performance of the mediators is presented in further mediation analysis.

#### Further Mediation Analysis

Further analysis shows the joint and separate effects of IT-c and OL on innovation performance of firms.

In Table 6, IT-c has a positive effect on innovation performance through its enhancement of OL, which in turn positively affects innovation performance. The indirect (average mediation effect) is positive and more than the direct effect. This further confirms that OL is highly significant but a partial mediator of the impact of IT-c on innovation performance (H4).

**Table 6:** Effect of IT-c on innovation performance through organisational learning

	Coefficient	Standard error	*z*	*p* >|*z*|	95% confidence interval	
Indirect effect: organisational learning						
Via research	1.499836 (0.418)	0.3945592	3.80	0.000	0.7265143	2.273158
Via experimentation	1.519267 (0.423)	0.4176103	3.64	0.000	0.7007654	2.337768
Via exploitation	0.3881816 (0.108)	0.2020745	1.92	0.055	− 0.0078771	0.7842402
Total indirect effect (organisational learning)	3.407284 (0.949)	0.606064	5.62	0.000	2.219421	4.595148

Total effect (3.589), given by total indirect/mediated effect and direct effect, where 3.407 and 0.182 are total indirect effect by all mediators (OL) and direct effect of IT-c, respectively. Proportion of total effect explained in parenthesis

Since there could be a violation of sequential ignorability assumption, in which case the error terms in the mediator-independent variable regression and full model are correlated,^5^ a sensitivity analysis was conducted to check the robustness of the estimate, following Hicks and Tingley (2011). Mediation sensitivity analysis helps to establish the reliability of the estimate by checking at what level of correlation between ε_*i*2_ and ε_*i*3_, denoted by *ρ*, the average causal mediation effect (ACME) would be zero. The value of *ρ* at which the ACME is zero was computed using OLS-based Structural Equation Mediation (SEM) sensitivity check (with 450 simulations), where IT-c is the treatment, variables of OL serve as the mediators, with innovation performance as the outcome.

**Fig. 1:**
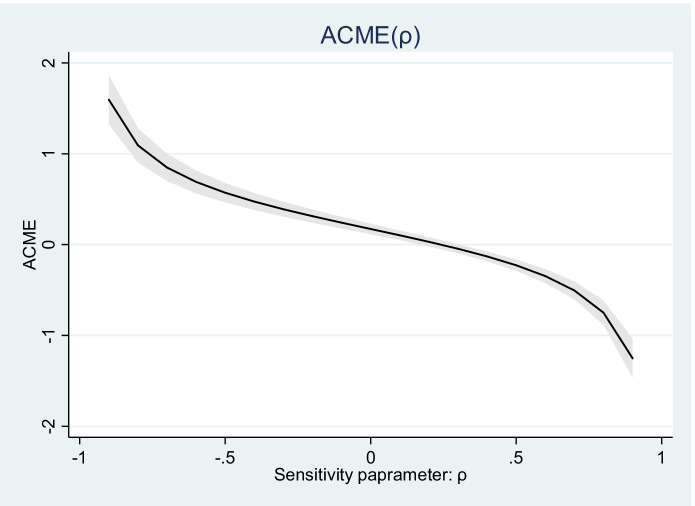
Sensitivity graph: the mediation role of organisational learning in the relationship between IT-c and innovation performance of firms

Based on the sensitivity analysis, for the estimate of ACME to be zero, the correlation (*ρ*) between the two error terms, ε_*i*2_ and ε_*i*3_, must be approximately 0.21, 0.31 and 0.11 for research-, experimentation-oriented and technology exploitation OL, respectively (Table 7; also shown in Fig. 1). Since the estimated ACME for the OL variables yields values that lie on the left hand of the estimated *ρ* thresholds, the estimates fall within the acceptance region: the mediation analysis is robust. IT capability indeed affects innovation performance through organisational learning, with a total indirect effect of 3.41 of the estimated 3.59 total effect. The indirect effect of IT-c via research-, experimentation-oriented and technology exploitation OL on the innovation performance of firms in Kenya jointly accounts for 94.9% (represented by the value 0.949) of the estimated total effect of IT-c (Table 6). Explorative OL (research and experimentation) accounts for a greater portion of the mediative role of OL in firms in this study than exploitative OL.

**Table 7:** Mediation sensitivity check

Organisational learning		
Research	Rho at which ACME = 0	0.215
Experimentation	Rho at which ACME = 0	0.313
Exploitation	Rho at which ACME = 0	0.108

95% confidence interval

### Discussion

This study examines the mediative role of OL in the relationship between IT-c and innovation performance of firms in Kenya. First, the results support the argument that the deployment of IT in business activities has significant positive effect on innovation performance of firms in Kenya. Second, OL, in form of internal knowledge re-(use) or technology adaptation, research and experimentation with new ideas in firms, significantly improves innovation performance. Linking these, it finds that IT-c enhances firms’ learning capabilities, which in turn increase innovation performance of firms.

This study contributes to understanding the OL channels of IT effect on firm innovation performance. It observed that experiment-oriented OL is a significant knowledge exploration mechanism through which IT-c influences innovation performance of firms in Kenya. The role of experimentation could be linked to the fact that experimentation is characterised by interaction and feedback, in which IT is key to enhancing creative initiatives amongst functional units via shared digital platforms (Benitez et al., 2018; Cenamor et al., 2019; D’Adderio, 2001). Research-oriented learning of firms in Kenya is another explorative OL link between IT-c and innovation performance. This indicates the role of research-related activities, and the fact that when added to the same equation as IT usage, the effect of research-related activities standouts compared to IT itself (Usai et al., 2021). In this context, IT is important, not in itself, but to the extent that it enables firms to improve research-related activities. This aligns with the reasoning that IT capability enables firms to improve knowledge acquisition and sharing. Searching for and sharing information enables the firm to improve product offerings and reconfigure its process to respond to market demand (Rialti et al., 2019; Tippins & Sohi, 2003).

This study finds that the intervening role of exploitative learning in the relationship between IT-c and innovation performance is less than the contribution of explorative learning routes (research- and experimentation-oriented OL). This may be partly explained by the fact that the firms investigated operate in a relatively turbulent environment, characterised by stiff competition, which drives exploration and innovation performance. The data presented earlier in this paper showed that informal competition, one of variables of business environment, tends to exert pressure on formal firms to innovate, given the difficulty of staying relevant in a market that has strong informality. It therefore follows, in line with literature, that where competition is stiff firms tend to depend on explorative learning to maintain their competitive advantage (March, 1991; Zack, 1999).

However, in an African context, some caution is needed when interpreting what constitutes research-oriented OL since much of the research-related activities of firms could be in form of search for technologies to imitate. This resonates with Oerlemans and Pretorius (2006) and Chan and Oerlemans (2011) who note that such firms in an SSA context innovate mainly through mimicking (technology search and copying). This gives credence to the positive relationship between IT-c and exploration (mainly through search for and domestication of external knowledge and technology, which in turn improve innovation performance of firms). Whilst copying is not necessarily a bad practice and such a case cannot be explicitly claimed in the findings for Kenya, it is important to place in context the high tendency towards explorative activities and the disproportionate intervening effect it has on innovation performance, as a mediator of IT-c, observed in this study.

## Conclusions and Reflections

This study sets out to explore the role that IT capability (IT-c) plays in firm innovation performance through the channel of organisational learning (OL) in Kenya. It establishes that IT-c has a significant effect on innovation performance of firms, enhancing their capability to simultaneously improve products, processes, organising and market development. This effect is mainly realised through the OL orientation of firms. It finds that the mediative role of OL in the relationship between IT-c and innovation performance is realised mainly through explorative learning in form of research-oriented and experimentation-oriented learning whilst enabling the firm exploit existing knowledge. The study has managerial and policy implications.

Top management who make decisions on investment in IT such as software acquisition, website development and other shared platforms and IT skills should think of integration with internal processes. IT-related investment and activities must take into account the competence and practices that encourage research, staff training and re-using knowledge for innovation. The business value of IT lies in its capability to enhance these activities and practices. Investing in IT infrastructure whilst keeping organisational culture that discourages new ideas can weaken the expected outcome.

In the aspect of policy, supporting information economy should look beyond IT infrastructure such as laying optic fibre and contribution of computers or building partnerships for such acquisitions. Such policies must complement IT uptake with skills necessary to implement it in firms. This could be in form of incorporating and strengthening the IT knowledge component in educational programmes so that it is easier for firms to hire IT-inclined people and invest less in retraining, at least for basic applications. Another important policy aspect is the need to pay attention to the role R&D and training can play in realising the gains of IT. The policy incentives can be in form of rebates given to firms that provide evidence for having conducted R&D and training.

This study encountered some limitations but has research possibilities. First, available data does not currently allow for longitudinal analysis, in which case lagged and time-consistent effects could have been observed. It therefore opens possibilities for follow-up research. In a related vein, the study can be built upon to investigate the role of OL in the relationship between IT-c and firm innovation performance across SSA. Second, the study captures IT capability in its basic sense, given the available data. IT-c itself corresponds to the level of development of countries; such that where technological advancement is still at the early stage, as in Kenya, the more advanced dimensions of IT applications would be limited. IT is a fast-paced phenomenon; hence, updated data is important to capture the most recent trends in firms. The technologies of now become obsolete later, with a possibility to deplete the value of previous IT-c, if the firm does not adapt. Third, and more of a research possibility, is that this study does not test the meditative role of the OL dimensions to yield different outcomes in terms of the quality of innovation. For example, it would be interesting to investigate whether and how IT-c, through explorative learning, contributes to superior innovation (novelty). Fourth, this study provides scope for follow-up data updates on and analysis of the role of IT capabilities and organisational learning in enhancing the resilience of firms in the unprecedented crisis presented by COVID-19 shocks.

## Appendix

**Table A1:** Principal components and variables of IT capability, including the excluded variable

Variable	(1)	(2)	(3)	Description
Email			0.40	Firm has an email
Website			0.55	Has a website
Software				Purchased software
Internal comm			0.31	Uses email/Internet for internal comm
Online purchase	0.49			Purchases online
Online sales	0.48			Sells online
Inventory	0.40			Keeps inventory of stock online
Online marketing	0.41			Uses Internet to conduct marketing
Online research	0.35			Uses Internet to conduct research
Mobile money		0.57		Uses mobile money
Mobile money 2				Uses mobile money (MM) to pay employees
Mobile money 3		0.43		Uses MM to pay suppliers
Mobile money 4		0.39		Uses MM to pay utilities bill
Mobile money 5		0.51		Use MM to receive payment from customers
IT specialist			0.34	Has some employees designated entirely to IT

Number of observations: 545. Rotation: orthogonal varimax (Kaiser off). Rotated components: blanks exclude loading < 0.3

**Table A2:** Principal components and variables of organisational learning

Variable	(1)	(2)	(3)	Description
Innovation training	0.49			Firm conduct staff training meant to innovate
RD	0.57			R&D (conducted by the firm; not outsourced)
Experimentation			0.57	Staff experimentation with new idea
Foreign licence			0.74	Firm holds a foreign technology licence
Market research	0.57			Uses the service of a market/consumer research consultancy
Existing design				Firm has a main innovative product that adapts existing in-house design
Existing process		0.72		Firm adapts existing process or technology already in use in-house

Number of observations: 781. Rotation: orthogonal varimax (Kaiser off). Rotated components: blanks are excluding loading < 0.4

## Footnotes

1 IT (also framed for operational purpose in this study as IT-capability, IT-c) refers to information collection, processing and exchange enabling resources in firms considered in three main dimensions: Internet-enabled infrastructure, human resources or skills to use the infrastructure and activities supported on the infrastructure.

2 Organisational learning is the process or activity through which a firm acquires, distributes and integrates knowledge. This is elaborated in the literature section.

3 This paper does not capture any measurements related to interpretation, which concerns the meaning of information as conceived by users.

4 The data was released in 2014. Therefore, all innovation-related measurements followed Oslo Manual (2005), not the 2018 version.

5 This could be due to unobserved factors and the tendency of firms that are innovative being the ones that deploy IT.
